# Covid-19 and some contours of India’s ongoing agrarian crisis

**DOI:** 10.1177/1942778620985194

**Published:** 2021-03

**Authors:** Shantanu De Roy, C. Saratchand

**Affiliations:** 1 210957 Department of Policy Studies, TERI School of Advanced Studies, Delhi, India; 2 Department of Economics, Satyawati College, University of Delhi, India

**Keywords:** agrarian crisis, Covid-19, government, MGNREGS, peasant, rural proletariat, crisis agraria, Covid-19, gobierno, MGNREGS, campesino, proletariado rural

## Abstract

The onset of the Covid-19 pandemic has led to an aggravation of the agrarian crisis in India. The rural proletariat, poor peasants, and a section of middle peasants in India have been adversely affected. The paper advances a composite policy initiative to deal with this aggravation of the agrarian crisis involving an expansion of the existing rural employment guarantee schemes, various input subsidies to farmers, universal provision of safety gear for rural producers, and an expanded public procurement of food grains. It concludes with the political prognosis of the proposed composite policy initiative.

## Abstract (es)

El inicio de la pandemia de Covid-19 ha provocado un agravamiento de la crisis agraria en India. El proletariado rural, los campesinos pobres y una parte de los campesinos medios de la India se han visto afectados negativamente. El documento presenta una iniciativa de política compuesta para hacer frente a este agravamiento de la crisis agraria que implica una expansión de los esquemas de garantía de empleo rural existentes, varios subsidios a los insumos para los agricultores, la provisión universal de equipos de seguridad para los productores rurales y una compra pública ampliada de granos alimenticios. Concluye con el pronóstico político de la iniciativa política compuesta propuesta.

## Introduction

The agrarian question^1^ in India underwent a change after independence (Patnaik, 1986). As was the case in most developing capitalist countries of the world after decolonization, redistributive land reforms did not take place in postindependent India, except in some pockets. Agrarian relations in India were decisively shaped by the capitalist mode of production in the domestic economy as well as in the world (involving mediation by other processes). This resulted in an agrarian setup whose features varied in different phases of postindependent India, but some broad trends stand out.

These *broad trends*,^2^ which are discernible in the agrarian setup of India, include the following. First, there has been an increase in peasant class differentiation and increase in the landlessness in the rural population and increasing rural–urban migration. But this process has involved the pauperization of the peasantry, resulting in their becoming workers in the informal sector (both in agriculture and elsewhere). Second, an increase in per capita food grain availability that happened earlier has been reversed in the period, since the nineties, when neoliberal reforms have proliferated in India. Third, there has been a decline in the ecological viability of agricultural production (in a spatially uneven manner) as conventional farming became more widely diffused. Fourth, there has been an increase in the technical composition of capital (a rise in the means of labor used per unit of output), particularly in agricultural production under the aegis of the rural rich. This increase was not matched by a more than proportionate increase in agricultural output. Therefore there has been a decline in agricultural employment. This process has gained momentum in the period when the neoliberal project has proliferated in India (Springer et al., 2016).

The onset of the Covid-19 pandemic and the policy response of the Indian state have resulted in a deepening of the agrarian crisis.^3^ The unplanned lockdown in India that has waxed and waned in different regions of India has resulted in multifarious disruptions in the reproduction process of agrarian relations. First, the unplanned lockdown has resulted in some cases in delays in sale of agricultural output by peasants, which has resulted in losses in case of perishable commodities. Public procurement of agricultural output has not been instituted to the required extent to alleviate this problem. Second, as a result of nonsale or partial sale of agricultural products at distress prices due to the unplanned lockdown, many peasants have been unable to meet debt payment commitments, which has led to a combination of pauperization or decreased or doubtful viability of future agricultural production (possibly for some middle peasants too apart from poor peasants) due to drying up of agricultural credit. Third, distress-based regional rural-to-rural or rural-to-urban migration, after the onset of the Covid-19 pandemic, has resulted in labor shortage for agriculture in some areas such as the Punjab. The rural rich have sought to respond to this by a wage freeze for local agricultural workers along with restrictions on labor mobility. In other words, there is an increase in the extent of formal unfreedom of the rural proletariat and poor peasants in these areas. Fourth, the unplanned lockdown has resulted in substantial distress induced urban-to-rural migration, which has resulted in a decline in both the living standards of the rural proletariat and poor peasants (including fresh entrants into these classes due to pauperization of other peasant classes) but also a possible decline in their bargaining power in wage negotiations. Limited expansion of the Mahatma Gandhi National Rural Employment Scheme (MGNREGS)-based employment has at best made a modest contribution in reversing this overall trend. Fifth, the income compression of the poor peasants and the rural proletariat due to the onset of the Covid-19 pandemic, along with gaps in food security on account of a targeted public distribution system, has resulted in rising food insecurity for members of these two classes and also a section of middle peasants. Sixth, the diffusion of Covid-19 infection to the rural areas on account of both the substance and details of the policy response to the Covid-19 pandemic has increased the disease vulnerability (both morbidity and mortality) of the rural population (especially the rural poor that comprises the rural proletariat, poor peasants, and a section of middle peasants).

The six dimensions of the process of aggravation of the agrarian crisis in India on account of the Covid-19 pandemic requires a composite political response, whose policy component is discussed in the next section of this paper. The policy response that is advanced here is not a comprehensive addressal of the agrarian question in India but a transitional policy platform that tackles the immediate crisis on account of the Covid-19 pandemic. The political feasibility of this transitional policy platform is discussed in the concluding section. Therein, possible (political) measures that could follow the transitional policy platform are also briefly examined.

## A transitional policy platform to deal with the agrarian crisis in India during the Covid-19 pandemic

The composite policy response to the six dimensions of the process of aggravation of the agrarian crisis, mentioned in the previous section, is first examined conceptually in this section. The accounting definition for profits for any farmer (poor peasant, middle peasant, rich peasant, and capitalist landlord) may be expressed as follows:

Profit = Gross Value of Output − Wage Bill − Interest Payments − Fertilizer Cost − Other Input Cost − Rent on Farm Land

Due to the Covid-19 pandemic, there have been adverse changes in all aspects of the different components of the expression of profit^4^ as discussed in the previous section. However, these adverse changes impact asymmetrically on different classes. For instance, capitalist landlords and rich peasants (collectively the rural rich) are often involved in other economic activities, which results in their being less adversely affected by the reduced viability of crop production. Besides, the rural rich often directly benefit from agrarian distress. This is the case since many of the rural rich double up either as private procurers of agricultural output, moneylenders, fertilizer, and other input dealers, or a combination of all of these. Thus, debt default by agricultural households or distress sale of agricultural output, for instance, benefits the rural rich.

Besides, rural proletariat and poor peasants are not able to obtain adequate rural employment as mentioned in the previous section. The prospects of middle peasants are possibly intermediate between the rural rich and rural proletariat and poor peasants.

The methodology of this paper involves a policy simulation exercise. The macroeconomic framework that underlies this policy simulation framework is of an economy that is divided into an agricultural and a nonagricultural sector. Working people in countries such as India tend to first try and meet their food requirement. Subsequently, their residual income is used for consumption of nonagricultural output. Thus, the magnitude and price of food (produced by the agricultural sector) impose a constraint on the demand for nonagricultural output. Import of food is possible but not to an extent (or at a price) that will overcome this constraint. Likewise, the existence of the public distribution system cannot overcome this constraint since it is neither universal in terms of coverage nor is the food issued per household adequate to avoid its purchase, by the working people, in the private market for food. Moreover, it has not been possible for economies such as India to achieve a magnitude of export of nonagricultural output that allows the abovementioned constraint to be overcome. We argue that public expenditure centered on the agricultural sector will have larger positive impacts on output and employment in the whole economy. In this light, we set out a series of interconnected policy proposals—namely, wage subsidy, fertilizer subsidy, credit subsidy, output price support, extension of employment guarantee, enhanced food security, and augmentation of rural public health.^5^ We discuss in the following subsections a calculation of the government budget allocation required to give effect to the composite policy response that encompasses the aforementioned seven components. We have used secondary databases on the Indian economy, which includes the National Sample Survey Office Reports, Census of India, Labour Bureau, Economic Survey of India, Reports of the Commission for Agricultural Costs and Prices, Reserve Bank of India, Budget documents of the Government of India, Food Grain Bulletin of the Government of India, MIS Reports of the Mahatma Gandhi National Rural Employment Guarantee Act, and several circulars of the Government of India for this policy simulation analysis.

### Wage subsidy

The sudden imposition of an unplanned lockdown in March 2020 severely disrupted commodity exchange in the rural areas. Ramakumar (2020) argued that except for maize, market arrivals for other crops were lower in 2020 as compared to 2019. He estimated that the market arrivals for the most important *Rabi* (winter) crop, wheat, were between one-half to three-fourths in 2020 of its corresponding level in 2019. Ramakumar (2020) also pointed out that the decline in wholesale price indices for cereals, rice and wheat is the most important food crops in India, vegetables, egg, and poultry chicken reflect low price realization for the farmers. The factors underlying this disruption included lack of adequate transportation. There were some labor shortages too, following the imposition of unplanned lockdown, enforcement of social-distancing norms and closure of regulated agricultural markets [*mandis*] due to inadequate health safety provisions. These have considerably slowed down procurement activities of *Rabi* season output in the *mandis,* resulting in economic losses for the poor peasants and possibly a section of middle peasants on account of long waiting times, perishable nature of many agricultural commodities, and lack of storage facilities with these classes. The rural rich, largely due to greater access over land, nonland inputs, and profits from nonagricultural activities, can not only withstand these shocks in the output market but also profit from them.

Two important features of the labor market in Indian agriculture are (a) pauperization of the peasantry whereby the rural proletariat and poor peasants participate as wage-labor to supplement their (poor peasants) meager incomes from crop production^6^; and (b) hiring of labor by different classes for various agricultural operations (e.g. Basole and Basu, 2011a; National Sample Survey Office, 2014a; Ramachandran, 2019). Disruptions in procurement of *Rabi* harvest are bound to adversely impact labor absorption (of hired labor) in agriculture, particularly for the poor and a section of middle peasants, during the upcoming *Kharif* (summer) agricultural season.

Thus, the rural labor market has been seriously disrupted due to factors such as: (a) “reverse migration” in the context of slowdown of rural economy and (b) disruption in exchange of agricultural output, which has had an adverse impact on income generation from crop production especially for the rural nonelite sections (poor peasants and possibly a section of middle peasants), thereby jeopardizing their ability to use wage-labor (as well as family labor) for the upcoming agricultural season. Modak et al. (2020) argued that the situation may not be as serious for the landed sections in the irrigated regions in India due to large-scale mechanization of agriculture.

We argue that providing wage subsidies to different sections of the peasantry (as well as other labor hiring classes) will enhance their ability to hire labor, generate employment in farm work, and may prevent real wages for agricultural work from falling and hence prevent the rural poor from being pushed below subsistence levels of consumption. We have used the National Sample Survey Office (National Sample Survey Office, 2014a)—Key Indicators of Situation of Agricultural Households in India for our analysis of the wage subsidy proposal.^7^ Data on average wage rate of agricultural work for male workers were obtained from the Labour Bureau (various years).

Total expenses on labor, average wage rate, and wage subsidy was estimated in 2019–2020 prices. Agricultural wage rate per day for male casual workers in 2019–2020 was Rs 291 (USD 3.94), and wage subsidy for each person day of employment was Rs 182 (USD 2.46), which is the average wage rate per person per day in MGNREGS work in 2019–2020. In other words, we are arguing that almost two-thirds (62.5%) of the wage rate will be subsidized by the state for each person day of labor used in agriculture. Based on this argument, total wage subsidy estimated was Rs 4702 crores, which was around 0.026% of the GDP in India in 2019–2020 (Table 1).

**Table 1:** Estimation of wage subsidy for crop production^8^

Selected features of agrarian economy in India	Estimation of wage subsidy
Total number of households involved in crop production (in crores)	8.3
Total expenses on labor (in Rs/2019–2020)	75,180,553,053
Average daily wage rate (men workers/in Rs/2019–2020)	291
Total person-days of labor used in agriculture	258,352,416
Wage subsidy (in Rs/2019–2020)	4702 crores
Share in GDP of 2020–2021 (%)	0.026

*Note*: GDP estimated in 2020–2021 is based on 10% decline in growth rate as compared to 2019–2020.

Average daily wage rate of men agricultural workers in January 2019–2020 has been used.

*Source*: National Sample Survey Office (2014a)—Key Indicators of Situation of Agricultural Households in India; Labour Bureau (various years); Economic Survey of India (2020).

### Fertilizer subsidy

According to the National Institution for Transforming India (NITI Aayog) of the Government of India, the agricultural sector is projected to grow at 3% in 2020–2021 (Centre for Monitoring Indian Economy, 2020). The Economic Survey of 2019–2020 estimated the gross value added (GVA) of agriculture and allied sector at Rs 3,450,623 crores. In 2020–2021, the GVA of agriculture and allied sector is projected to be Rs 3,554,142 crores (with 3% growth rate). According to the Union Budget documents, fertilizer subsidy consists of two components: (a) urea subsidy and (b) nutrient-based subsidy (Ministry of Chemicals and Fertilizers, 2020). Share of fertilizer subsidy in GVA of agriculture and allied sector in 2019–2020 was 2.3%. However, in the Union Budget of 2020–2021, budget allocated on subsidies was Rs 71,309 crores, which is almost Rs 8600 crores lesser than the revised estimates in 2019–2020. Based on the projected growth rate of agricultural sector in 2020–2021, the share of subsidies in GVA of agriculture and allied sector in 2020–2021 has been reduced to 2%.

We argue that in view of the deepening of the agrarian crisis following the Covid-19 pandemic, the share of subsidies in GVA of agriculture and allied sectors should not be reduced in 2020–2021 as compared to 2019–2020, to maintain viability of agricultural production, especially for the poor peasants and a section of the middle peasants. In other words, we are arguing that the share of fertilizer subsidy in the GVA of agriculture and allied sector should be maintained at 2.3% in 2020–2021. Based on this, the estimated budget allocation for providing subsidy on fertilizers is Rs 82,398 crores in 2020–2021, almost Rs 11,000 crores higher than the actual budget allocation.

### Indebtedness and credit subsidy

There are certain aspects of the rural credit market in India that are noteworthy. First, typically, access of the rural poor to formal credit institutions is relatively limited when compared to the rural rich (Ramachandran, 1990). Hence, the rural poor are compelled to depend on moneylenders to meet credit requirements; second, even with limited access to formal sector credit, size of loans and hence amount borrowed by the poor forms a small part of their total borrowing; third, by and large, the rural poor borrow to meet (often subsistence) consumption needs, while the rich take loans for production.

**Figure 1:**
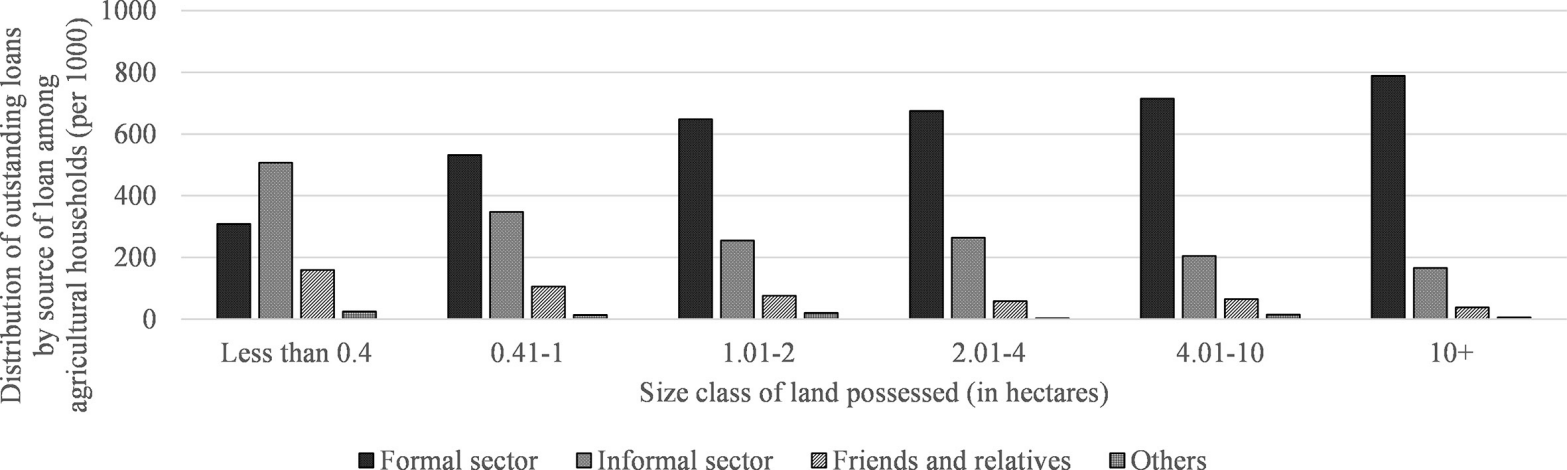
Distribution of agricultural households by source of loans for each size class of land possessed in India.

With the further proliferation of the neoliberal project in the 1990s, there were changes in policies of commercial banks related to credit disbursals, whereby banking (which was aligned to the dirigiste regime) now was enmeshed in the process of financial liberalization. Public sector banks too were oriented toward according primacy to profit-based activities. As a result, as Ramakumar and Chavan (2007) and Ramakumar (2019) argue, there has been a de-facto greater exclusion in terms of credit of the rural poor. Inequity in access to formal sector loans can be clearly seen in Figure 1, which shows that access to formal credit is inextricably related to access to land—higher the extent of land possessed, greater is the access to loans from formal credit institutions.

As agricultural exchange was disrupted due to the enforcement of unplanned lockdown, incomes of the rural poor were adversely affected. Hence, their requirement of affordable credit for cultivating *Kharif* crops increased substantially. In this context, it is crucial that access to formal credit institutions of these sections of the peasantry, hitherto excluded, is adequately enhanced. In this paper, we argue for providing credit subsidy to ward off the crisis emanating from the Covid-19 pandemic.

Credit subsidy in the agricultural sector is estimated as the difference between interest earned and the cost of supplying loans to the sector. We argue that subsidized credit be provided to the crop producers through the Kisan Credit Card (KCC) scheme of the Reserve Bank of India, which is disbursed through various public sector and private banks. Based on the estimations of the Agricultural Credit Review Committee (ACRC), Sahu and Rajasekhar (2006) mentioned three broad categories of costs—financial, transaction, and costs of bad debts or risk costs—for loans disbursed by formal credit institutions.^9^ Total estimated cost incurred by the commercial banks for credit disbursal was Rs 14.5 for every Rs 100 of loan disbursed, and average interest rate on loans disbursed under the KCC scheme was 4%. Thus, subsidy for every Rs 100 of loan disbursed was Rs 10.5 (bankbazaar.com, 2020a).

We argue for extending the KCC scheme to eliminate the gap in its coverage by incorporating 7.9 crore holdings (households), three crore beneficiaries of Pradhan Mantri Kisan Samman Nidhi (PM-KISAN) scheme who do not have KCC (Department of Agriculture, Cooperation and Farmers Welfare, 2020). We also argue for extending the benefits of this scheme for the landless and other tenant farmers who have access to tiny plots of land (lesser than 0.004 hectares). Most of these poor peasants may not have access to credit from formal institutions. This will be the case since formal lenders particularly commercial banks will insist on collateral when they are incorporated into the process of financial liberalization. Total number of households in this category as estimated by the National Sample Survey Office (2014a) was 2.7 crores. In all, we argue that the scheme of providing subsidized credit needs to be extended to 13.6 crore households who hitherto have been left out. Based on the estimations of the Reserve Bank of India (2017), we argue for a credit limit of Rs 1.33 lakh for each beneficiary.^10^ In this sense, we argue for a minimum level of government intervention in the credit market in rural India that can provide a modicum of relief to substantial sections of the population in rural India. Table 2 gives the detailed breakup of estimating credit subsidy to rural cultivators and shows that about 1% of the GDP of 2020–2021 has to be allocated to provide subsidized credit to the suggested beneficiaries.^11^

**Table 2:** Estimation of credit subsidy for crop production

Selected features of credit disbursals in India	Estimation of credit subsidy
Estimated lending costs by the commercial banks for every Rs 100 of credit disbursed (in Rs)	14.5
Estimated interest income earned by the commercial banks for every Rs 100 of credit disbursed (in Rs)	4
Extent of credit subsidy (in Rs)	10.5
Total number of additional beneficiaries under the KCC scheme	13.6 crores
Minimum limit for disbursal of credit for each beneficiary (in Rs)	1.33 lakhs
Total amount of credit to be disbursed (in Rs crores)	1,803,154 crores
Extent of credit subsidy (in Rs crores)	189,331.2 crores
Share in GDP (2020–2021/in %)	1.02

*Note*: GDP estimated in 2020–2021 is based on 10% decline in growth rate as compared to 2019–2020.

*Sources*: Economic Survey of India, RBI-Revised Scheme for Issue of KCC, Sahu and Rajasekhar (2006), NSSO—Employment and Unemployment Situation in India (2014b), Department of Agriculture, Cooperation and Farmers Welfare (2020).

### Price support in the output market

Price support in the output market and procurement of agricultural crops serve the dual purpose of (a) increasing viability of crop production especially for the poor and middle peasants by way of which they are more likely to enhance production and undertake investment on land and non-land inputs that are considered to be crucial for enhancing labour productivity and land augmenting technical change in agriculture and (b) with increased production, stocks of food grains with the government increases, that, in turn, can be allocated through the public distribution system (PDS) at subsidized prices. Thus, providing remunerative prices to the farmers during procurement and distributing food grains at subsidized prices through the PDS, are inextricably related and crucial in terms of providing food security to substantial sections of the working population in India.

Even though Kannan (2015), NITI Aayog (2016), and De Roy (2018) argue that the provision of support prices in India are beset with problems, these are necessary for the reproduction of agrarian India. In this paper, we argue for extending the reach of government in procurement of agricultural crops in India. This assumes importance in view of the recent studies by Rawal et al. (2020) and Rawal and Verma (2020), which showed that disruptions in agricultural exchange, following spread of the Covid-19 pandemic and subsequent unplanned lockdown, have compelled (poor) cultivators to sell crops at prices that are lower than the previously prevailing levels of minimum support price (MSP) for wheat, the most important *Rabi* crop. This has been corroborated by Azim Premji University (2020), which revealed that substantial sections of the farmers (70%–90%), across Indian states, were unable to sell their produce at MSP or above during the lockdown period.

We argue for the extended reach of the government in marketing in terms of:

1. Estimating cost of cultivation of each of the crops, based on the C_2_ cost concept rather than the officially used A_2_ +FL.^12^
2. MSP for each of the crops is estimated with a profit margin of 50% on C_2_, based on the proposals of the NCF and the High-Level Committee.
3. MSP to be given to the farmers for each of the crops—20 in all—as recommended by the Commission for Agricultural Costs and Prices (CACP) during *Kharif* and *Rabi* seasons^13^; in practice, MSP is by and large provided for rice and wheat.
4. Offer to procure up to 75% of the total production of each of the crops for which public procurement has been recommended. It can be argued that procuring 75% of output of food grains, including staples like rice and wheat, from the poor peasants and possibly middle peasants may have an adverse impact on their food security. However, the risk of food insecurity can be countered by an effective and near universal public distribution system (involving self-selection of beneficiaries and therefore no exclusion errors). Apart from this, the increases in income due to the price support measures would also increase the income of cultivators. This increase would be greater if the coverage of MSP is extended in terms of number of crops that are covered and use of the suggested cost concept used for estimating it. Tables 3 and 4 show the extent of proposed government intervention in agricultural price support.

**Table 3:** Recommended minimum support prices (MSPs) and projected state expenditure on procurement, *Rabi* crops, 2020–2021

Crops	Cost of cultivation (2019–2020) (Rs/quintal)		Cost of cultivation (2020–2021) (Rs/quintal)		MSP (50% over C_2_) (Rs/quintal)	Growth rate of MSP over 2019–2020 (%)	Total production (million tons)	Output procured (million tons)	Total expenditure by the government on procurement
	A_2_ + FL	C_2_	A_2_ + FL	C_2_					
Wheat	923	1425	984	1517	2275	18.2	107.2	80.40	1,820,000,000,000
Barley	919	1347	982	1455	2183	43.1	1.73	1.30	2,831,831,208
Gram	2801	4023	2975	4217	6325	29.8	10.1	7.58	47,914,724,013
Lentil	2727	4286	2937	4358	6537	36.2	1.6	1.20	7,844,752,740
Rapeseed and mustard	2323	3401	2440	353020	5295	19.7	8.78	6.59	34,864,533,682
Safflower	3470	4593	3655	5181	7771	49.0	0.02	0.02	155,419,810.9
Total expenditure by the government									275,611,261,455

*Notes*: Total production of wheat is for 2019–2020 as obtained from Food Grain Bulletin, 15 May 2020.

Total production of other crops is for 2018–2019 as obtained from the Reports of the Commission for Agricultural Costs and Prices (CACP), 2020–2021 (*Rabi* marketing season).

Projected cost of cultivation of crops in 2020–2021 was estimated. The estimation was based on the assumption that the growth rate of increase in the cost of cultivation for each of the crops between 2018–2019 and 2019–2020 is maintained between 2019–2020 and 2020–2021 in A_2_+ FL and C_2_.

GDP in 2020–2021 has been estimated based on the assumption that there will be decline in nominal growth rate by 10% in 2020–2021 as compared to 2019–20.

*Sources*: Food Grain Bulletin, Department of Food and Public Distribution (2020); Commission for Agricultural Costs and Prices (2019a), Rabi marketing season, 2020–2021.

**Table 4:** Recommended minimum support prices (MSPs) and projected state expenditure on procurement, *Kharif* crops, 2020–2021

Crops	Cost of cultivation (2019–2020) (Rs/quintal)		Cost of cultivation (2020–2021) (Rs/quintal)		MSP (50% over C_2_) (Rs/quintal)	Growth rate of MSP over 2019–2020 (%)	Total production (million tons)	Output procured (million tons)	Total expenditure by the government on procurement
	A_2_ + FL	C_2_	A_2_ + FL	C_2_					
Paddy	1208	1619	1251	1680	2520	37.3	117.94	88.5	222,933,000,000
*Jowar*	1698	2324	1781	2474	3711	44.4	3.7	2.8	10,298,567,790
*Bajra*	1083	1463	1185	1617	2425	17.5	8.51	6.4	15,477,067,856
*Maize*	1171	1570	1212	1665	2498	29.5	27.82	20.9	52,124,609,160
*Ragi*	2100	2672	2284	3012	4519	30.3	1.21	0.9	4100,646,924
*Arhar (Tur*)	3636	5417	3852	5891	8836	34.4	3.5	2.6	23,195,763,281
*Moong*	4699	6359	4748	6563	9845	39.6	2.37	1.8	17,498,929,098
*Urad*	3477	5460	3516	5975	8963	57.2	3.21	2.4	21,578,749,920
Groundnut	3394	4352	3533	4525	6787	33.3	6.5	4.9	33,087,412,800
Soybean	2473	3422	2699	3940	5910	59.3	13.74	10.3	60,903,953,541
Sunflower	3767	4957	3950	5459	8189	44.9	0.2	0.2	1,228,307,423
Sesamum	4322	6125	4484	6198	9297	43.4	0.75	0.6	5,229,467,578
Nigerseed	3960	5913	4002	6809	10,213	71.9	0.06	0.0	1,021,322,925
Cotton	3501	4678	3570	4848	7272	38.4	27.59	20.7	150,470,006,092
Total expenditure by the government									619,147,804,388

*Notes*: Total production of wheat is for 2019–2020 as obtained from the Food Grain Bulletin, 15 May 2020.

Total production of rice in 2019–2020 is the aggregate of Kharif and Rabi seasons. This is obtained from the Food Grain Bulletin, 15 May 2020.

Total production of other crops is for 2018–2019 as obtained from the CACP Report, 2019–2020 (Kharif marketing season).

Cost and MSP of paddy (common), jowar (hybrid), and cotton (medium staple) have been considered for this analysis.

Projected cost of cultivation of crops in 2020–2021 was estimated. The estimation assumed that the growth rate of increase in the cost of cultivation for each of the crops between 2018–2019 and 2019–2020 is maintained between 2019–2020 and 2020–2021 in A_2_ + FL and C_2_.

GDP in 2020–21 has been estimated based on the assumption that there will be decline in nominal growth rate by 10% in 2020–2021 as compared to 2019–2020.

*Sources:* Food Grain Bulletin, Department of Food and Public Distribution (2020); Commission for Agricultural Costs and Prices (2019b), *Kharif* marketing season, 2019–2020.

In all, government expenditure is Rs 89,476 crores, of which Rs 61,915 crores needs to be allocated for procuring *Kharif* crops for which MSP has been recommended. We estimate that the government will have to allocate around 3.9% of agricultural GVA and 0.5% of the (estimated) GDP of 2020–2021 to expand its intervention in agricultural price support. We argue that extended intervention by the government in procurement of agricultural crops will need to be supported with increased government intervention in agricultural marketing, particularly in the creation of marketing and warehousing infrastructure in India, with “multiplier” impacts on the rural economy.

### Extension of wage work: MGNREGS and Garib Kalyan Rozgar Abhiyaan (GKRA)

**Figure 2:**
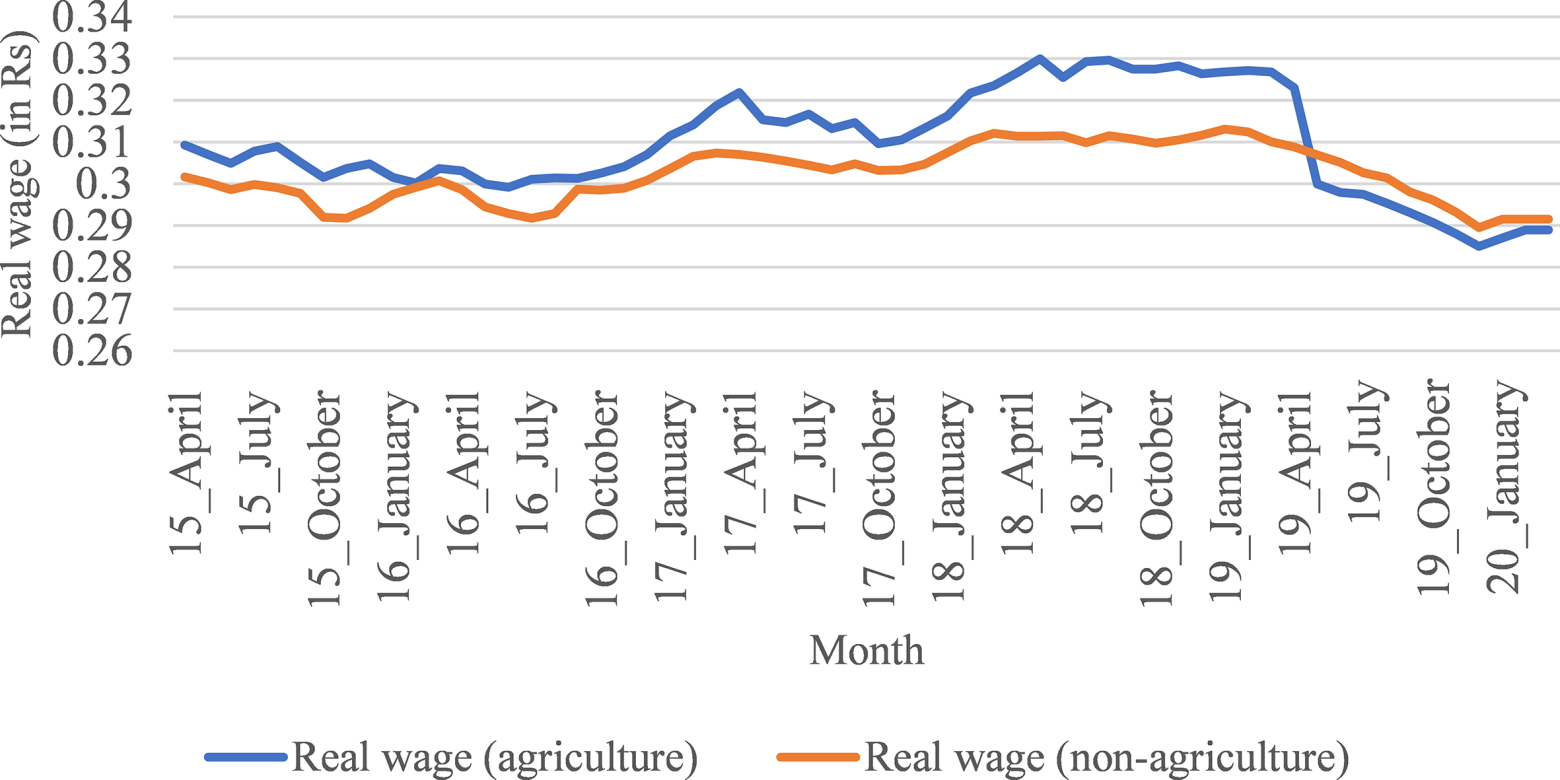
Trend in real wage rates in agriculture and nonagricultural workers (men), from April 2015 to March 2020 (in Rs).

**Figure 3:**
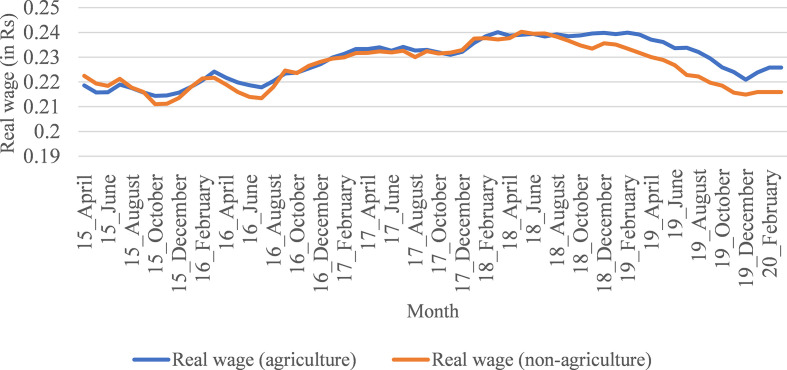
Trend in real wage rates in agriculture and nonagricultural workers (women), from April 2015 to March 2020 (in Rs).

“Reverse migration” of (primarily informal sector) workers from large urban areas areas to rural areas and small towns took place following suspension of economic activities in the former due to the unplanned lockdown. This is bound to have adverse consequences in terms of transmission of Covid-19 in the rural areas that had been relatively less affected in the early phase of the pandemic in India. Moreover, this reverse migration will have a negative impact on the real wage of the rural proletariat and poor peasants. Prior to the emergence of the Covid-19 pandemic, real wages were stagnant for a significant period of 2018. Subsequently, rural real wages have started to decline since the last quarter of 2018. The decline in rural real wages has been greater since 2019 (Figures 2 and 3). In this context, return of the migrant workers will lead to additional downward pressure on real wages, other things remaining the same, and further contraction in rural demand for necessary means of consumption.

**Figure 4:**
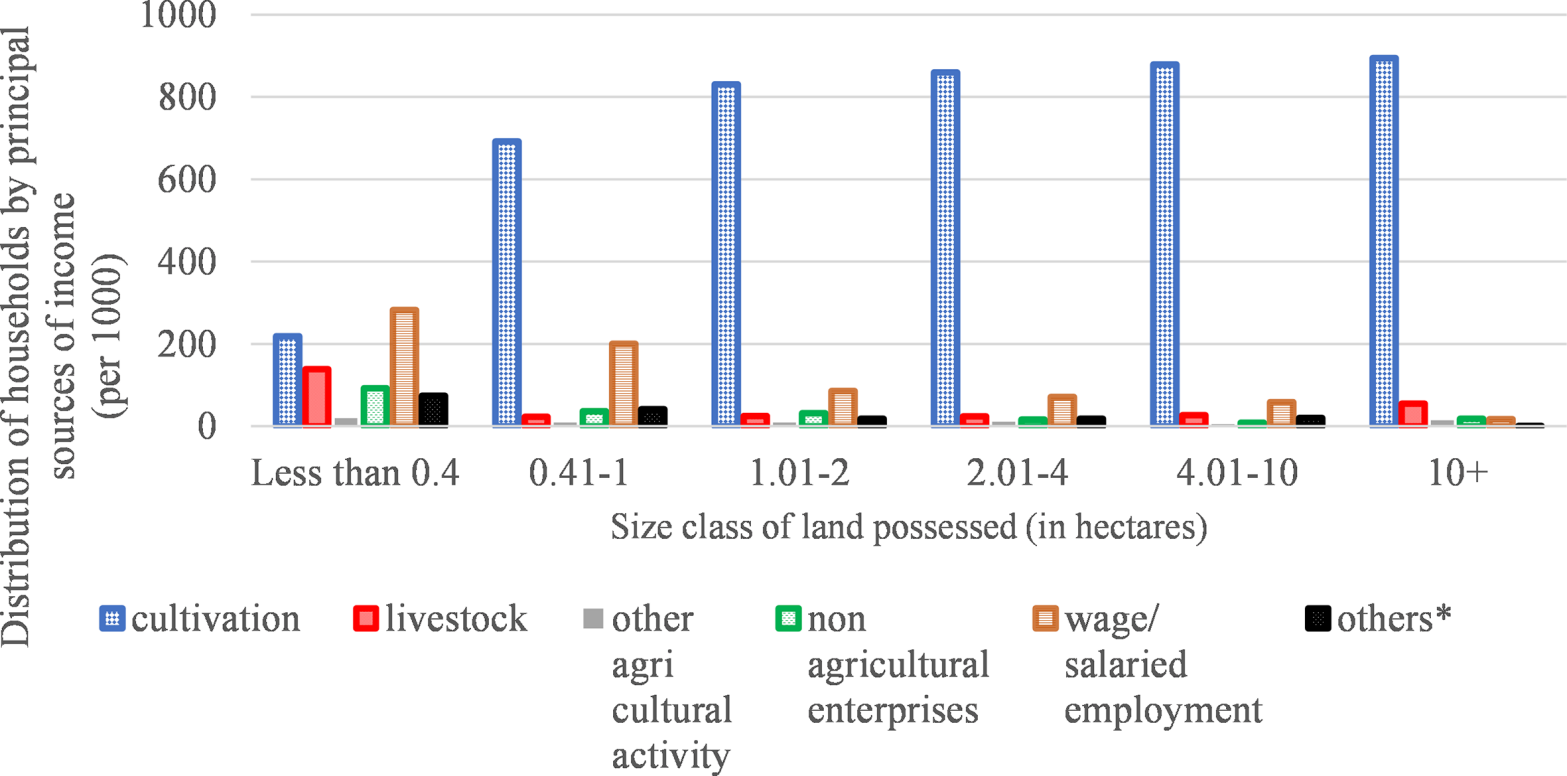
Distribution of agricultural households by principal source of income for each size class of land possessed.

National Sample Survey Office (2014a) has clearly established that wage work is a crucial livelihood source for agricultural households possessing not more than one hectare of land.^14^ In contrast, cultivation of agricultural crops is the principal source of income for majority of households that possessed more than one hectare of land (Figure 4). Figure 4 demonstrates a trend of declining importance of wage incomes with increased access to land. Reverse tenancy would further exacerbate this trend.

There is extensive literature, based on primary data, that shows increasing importance of nonfarm employment in rural India in terms of enhancing incomes of the rural poor who are oppressed (e.g. Basole and Basu, 2011a; Heyer, 2019; Himanshu, 2011; Lindberg, 2019; Rawal et al., 2019). Studies have also revealed the existence of exploitative tenancy and labor relations due to landlessness and lack of gainful wage employment, outside of agriculture in rural India (e.g. Rawal, 2006; Rawal and Osmani, 2009).^15^ Thus, proliferation of nonfarm (primarily informal sector) employment in India can be associated with both “pull” and “push” factors in the urban and rural areas, respectively.^16^ Based on the National Statistical Office (2019)–Periodic Labour Force Survey database and the National Sample Survey Office (2014b) Employment-Unemployment Survey, Rawal et al. (2020) argued that there was a decline in work participation rates for men and women workers, which would have adversely impacted the rural proletariat, poor peasants, and a section of middle peasants, sections of the rural population for whom wage work is important. Moreover, the economic slowdown has led to a sharp decline in real wages since the last quarter of 2018 (Figures 2 and 3).

After the Covid-19 pandemic materialized and was transmitted through various parts of India, health and economic crises emerged with the process of “reverse migration,” referred to above. With many large urban areas under various types of lockdown, a significant source of employment was lost for workers who had migrated to these areas (and were primarily located in the urban informal sector). The adverse consequences for the rural poor of the unplanned nature of the lockdown were compounded by its timing. Since July 2020, when some have argued for relaxations in various lockdown initiatives, the prospects of return of the migrant workers to their previous jobs in large urban areas—which were the first epicenters of the pandemic in India where there is de-facto community transmission of Covid-19—appear to be dim due to: (a) inadequate arrangements of health and safety arrangements for these workers who are mostly employed with the informal sector with a virtual absence of social security arrangements and (b) reduced ability of most urban firms especially the micro, small, and medium enterprises (MSMEs), in the absence of adequate government support, to employ workers due to losses incurred during the lockdown. According to the database of the Centre for Monitoring Indian Economy (2020), rural workforce declined by 9 million (264 to 255 million) between January–April 2020 and May–August 2020; the corresponding figure for urban areas was 1 million (117 million to 116 million). During this period, unemployment rate in the rural areas increased by 10.7%, while in the urban areas it has remained unchanged. The database has revealed that decline in gainful employment opportunities was more in the rural areas than their urban counterparts. Thus, with an enlarged reserve army of labor, rural real wages that were already showing a sharp declining trend since 2018 are likely to remain depressed for quite a while.^17^

In this context, we argue that the MGNREGS can turn out to be crucial in increasing the bargaining power of the rural poor which would also play a pivotal role in arresting the declining trend of rural real wages. On 20 April, the union government through a notification exempted wage work under the MGNREGS from lockdown restrictions. In this paper, we argue that extension of the MGNREGS and the recently introduced Garib Kalyan Rozgar Abhiyaan (GKRA) are two such policy initiatives that may be undertaken, along with the modifications suggested herein, by the state to overcome the crisis confronting the rural workforce.

MIS Reports of the MGNREGS (2020) show that all households that demanded work under the scheme did not receive work. In 2019–2020, about 11% of the households did not receive work under MGNREGS, despite demanding it. The corresponding figure for 2020–2021 is 19% till the end of June 2020. In this paper, we argue for the extension of the MGNREGS by analyzing four different scenarios. This is shown in Table 5.

**Table 5:** Patterns of expanding wage work under the MGNREGS and GKRA

MGNREGS	
**100 days of employment are received by each of the households that are provided wage work under the Act**	
Total number of households that received work	44,902,214
Average wage rate per day per person (2020–2021/in Rs)	183
Wage bill (in Rs)	82,171.05 crores
Material and administrative cost (in Rs)	41,085.5 crores
Total expenditure (in Rs)	123,256.6 crores
Share in GDP of 2020–2021 (%)	0.66
**100 days of employment are received by each of the households that demanded wage work**	
Total number of households that demanded work	55,344,512
Average wage rate per day per person (2020–2021/in Rs)	183
Wage bill (in Rs)	101,280.5 crores
Material and administrative cost (in Rs)	50,640.25 crores
Total expenditure (in Rs)	151,920.8 crores
Share in GDP of 2020–2021 (%)	0.82
**100 days of employment are received by each of the households that demanded wage work at the legal minimum wage**	
Total number of households that demanded work	55,344,512
Legal minimum wage rate (in Rs)	403
Wage bill (in Rs)	223,038.4 crores
Material and administrative cost (in Rs)	111,519.2 crores
Total expenditure (in Rs)	334,557.6 crores
Share in GDP of 2020–2021 (%)	1.81
**200 days of employment are received by each of the households that demanded wage work at legal minimum wage**	
Total number of households that demanded work	55,344,512
Legal minimum wage rate per person per day (in Rs)	403
Total wage bill (in Rs)	361,911.8 crores
Material and administrative cost (in Rs)	180,956 crores
Total expenditure (in Rs)	542,867.8 crores
Share in GDP of 2020–2021 (%)	2.95
GKRA	
Number of migrant workers to be covered	2.8 crore
Days of employment	250
Legal minimum wage rate per person per day (in Rs)	403
Wage bill (in Rs)	282,100 crores
Material and administrative cost (in Rs)	141,050 crores
Total expenditure (in Rs)	423,150 crores
Share in GDP of 2020–2021 (%)	2.3

*Notes*: Average wage under MGNREGS was estimated by using the wage rates in each of the states in 2020–2021.

GDP of 2020–2021 was estimated based on the assumption that growth rate of GDP in 2020–2021 will decline by 10% as compared to 2019–2020.

Legal minimum wage rate of construction work has been considered. GKRA, Garib Kalyan Rozgar Abhiyaan; MGNREGS, Mahatma Gandhi National Rural Employment Scheme.

*Sources:* Chief Labour Commissioner (Central) (2020); Ministry of Finance (2020); Ministry of Consumer Affairs Food and Public Distribution (2020d); MGNREGS (2020)

One of the biggest criticisms of MGNREGS is its inability to create adequate magnitude of employment in rural India since like all other policies, it has been incorporated within the ambit of the neoliberal project in India. In 2019–2020, only 6.5% of households that demanded wage work under MGNREGS received employment for the full 100 days. Moreover, the average wage rate under the scheme is lower than the legal minimum wage rate in other nonagricultural occupations and wage rate of agricultural workers.

Table 5 sets out the details of the proposed expansion of the MGNREGS in terms of: (a) 100 days of guaranteed employment for each of the households that received work under the scheme, (b) 100 days of guaranteed employment for each of the households that demanded work, (c) 100 days of guaranteed employment for each of the households that demanded work under the scheme at the legal minimum wage, and (d) 200 days of guaranteed employment for each of the households that demanded wage work at the legal minimum wage. The 4-year (2016–2017 to 2019–2020) average share of expenditure on material and administration in total expenditure was about one-third (32%; MGNREGS, 2020). This implies that roughly around half of the wage bill is incurred as material and administrative expenses. In this analysis, material and administrative costs are estimated as half of the wage bill.

Table 5 provides information on the resources that need to be allocated. The quantum of resources required for this purpose is quite modest and amounts to not more than 2.95% of GDP when the scheme is expanded to 200 days of guaranteed employment for each household that demand wage work at the legal minimum wage rate. Needless to say, only households who belong to the rural proletariat, poor peasants, and a section of middle peasants are likely to make use of this proposed expanded variant of MGNREGS.

In the month of June 2020, the Government of India announced the initiation of a “new” scheme, namely the GKRA, “to boost employment and livelihood opportunities for migrant workers returning to villages in the wake of Covid-19 outbreak” (see the Prime Minister’s Office, 2020).^18^

Some salient features of this scheme include:

- Initiated for the returnee migrant workers and rural citizens in 116 districts located across Bihar, Uttar Pradesh, Madhya Pradesh, Rajasthan, Jharkhand, and Odisha.
- Intends to provide employment for 125 days under 25 categories of public work that includes construction of roads, Anganwadi Bhawans, houses, panchayat offices, community toilets, rural *mandis*, and other durable rural infrastructure.
- Rs 50,000 crores has been allocated for this scheme.^19^

In this paper, we argue that in view of the severity of the ongoing crisis in India, an expansion of the GKRA is called for along the following lines: (a) increase in the number of days of employment to 250 from the current level with a 5-day working week; (b) incorporation of all returnee migrant workers (through a process of self-selection) and in that way the geographical spread of the GKRA can encompass the whole of the country; (c) providing statutory legal minimum wage to the workers (enrolled in the GKRA). However, the current text of the GKRA is noncommittal in this regard.

We argue, at the very least, for implementing the expanded version of the GKRA for the financial year of 2020–2021. The union government in a press release on 2 July had projected a figure of 2.8 crore migrant workers in India (Ministry of Consumer Affairs Food and Public Distribution, 2020a).^20^ We argue that expanding the ambit of this scheme for 2.8 crore migrant workers—almost half of the projected figures for 2020 (based on extrapolation of the relevant figure in Registrar General and Census Commissioner, 2011) of 5.55 crores—will enhance incomes of migrant workers in all areas of India. We have also used the statutory legal minimum wage for estimating the financial allocations required for the expanded variant of the GKRA. Material and administrative costs incurred under the GKRA are half of the total wage bill, as seen in Table 5. This has been estimated based on the ratio of expenditures on labor and other items (material and administrative) under the MGNREGS in which wage component is double the latter. Table 5 shows that the wage bill is lesser than the prescribed allocation under this scheme and not more than 2.3% of the projected GDP of 2020–2021 needs to be allocated.

### Tackling food (in)security: Extending the ambit of National Food Security Act, 2013

Based on the National Sample Survey Office (NSSO) consumer expenditure survey of 2011–2012, Swaminathan (2019) estimated that 95% of households in rural India and 90% in urban India are food-insecure. Her findings are based on the food share criterion in which households that spend at least a third of total consumption expenditure on food are poor households. In view of the existence of widespread hunger, as reflected in the latest consumer expenditure survey report, a government committed to eradicating food insecurity would have acted to overcome this through an authentic universalization of the PDS.^21^

In this paper, we argue for universalizing the public distribution system in the rural areas, at least for the financial year of 2020–2021. Among others, Banerjee (2011), Patnaik (2003), and Swaminathan (2008) have argued for universal PDS in India. The demand for universalizing access to food has gained momentum in view of the multipronged crisis that has been unfolding after the emergence of the Covid-19 pandemic (see Sinha, 2020: Bhattacharya, 2020; Rajan et al., 2020).

The National Food Security Act (NFSA), 2013 seeks to cover 75% of the rural population and 50% of the urban population in India under the Antyodaya Anna Yojana (AAY) and priority households (see Ministry of Consumer Affairs Food and Public Distribution (2020b and 2020c).^22^ But the implementation of the NFSA has not always met these stipulations. In other words, there have been many instances of slippages in this respect.

In this paper, we argue that the ambit of NFSA in the rural areas be extended to all households (through a process of self-selection). In other words, we suggest that 35 kg of food grains be made available to every household in the rural areas under the AAY. To that end, we argue for allocation of 13 kg each of rice and wheat, 4 kg of pulses, and 5 kg of coarse grains (millets) per month. This implies that in a family comprising four persons, on average, each of them will be receiving 108.3 grams each of rice and wheat, 33.3 grams of pulses, and 41.7 grams of coarse grains per day. Based on FAO (2003) estimates of energy conversions for various food items, we argue that allocations of rice, wheat, pulses, and grams, as per the suggested quantity, will enable members of each of the households in rural areas to exceed minimum calorie requirement—2400 Kcal/per capita/per day; thereby, they can be food-secure.^23^ It can be assumed that the rural rich (capitalist landlords and rich peasants) will self-select out of the system to avoid queuing time and related factors including existence of alternative options to obtain access to food grains.

Table 6 shows, in detail, the allocation of food grains and estimated subsidy that needs to be provided by the government for universal provision of food grains through the PDS. Total allocation of food grains under the (extended) NFSA is estimated based on the number of rural households in 2020 projected according to the data of the Registrar General and Census Commissioner (2011). The government will incur subsidies in (a) allocating food grains at a reduced price and (b) carrying cost in terms of storage and handling of food grains. It is expected, not implausibly, that the quantum of subsidy incurred for storage and handling and hence total subsidy will reduce if an authentic universal PDS is extended to the urban areas (which would directly enhance the consumption of workers and petty producers).

**Table 6:** Quantum of allocation of food grains through universal PDS under the Antyodaya Anna Yojana (AAY) in the rural areas and subsidy cost of the government

Crops (1)	Procurement (in million tons) (2)	Allocation for the NFSA		Excess stocks (total procurement-total allocation under the NFSA) (in million tons) (5)	Quantum of subsidy for issuing through the PDS (in Rs)		Quantum of subsidy for storage, handling (in Rs)		Total subsidy (in Rs crore) (7 + 9)
		Per household per month (in kg) (3)	Total allocation in 2020–2021 (in million tons) (4)		Per kg (MSP/kg-issue price/kg) (6)	Total (in crore) (7)	Per kg (8)	Total (excess stocks * cost incurred per kg (in crore) (9)	
Wheat	80.4	13	36.1	44.3	20.75	74,890.7	26.84	118,901.2	193,791.9
Rice	88.5	13	36.1	52.4	22.2	80,124	37.27	195,127.1	275,251.1
Pulses	15.6		11.1	4.5	60.25	66,908.8	32	14,400	81,308.8
Coarse grains	29.5		13.9	15.6	24	33,315.6	32	49,920	83,235.6
Total	214	35	97.2	116.8	NA	255,239.1	NA	378,348.3	633,587.4
Share in GDP of 2020–2021 (%)									3.44

*Notes*: Pulses consist of gram, lentil, *arhar (tur*), *moong, urad*. Coarse grains consist of barley, *bajra*, maize, and *ragi*.

Projected rural population in 2020 according to the Census of India 2011 database was 925,433,227.3 crore. Assuming average household size of four, total number of rural households in 2020 is 231,358,306.8 crores.

Quantum of subsidy for storage, handling of pulses and coarse grains, per kg, is the simple average of corresponding figures of rice and wheat.

MSP of gram and maize have been considered since these are mostly widely produced crops among pulses and coarse grains. Pulses are not distributed under the NFSA. We have considered the issue price of pulse at Rs 3/kg.

GDP in 2020–2021 has been estimated based on the assumption that there will be decline in nominal growth rate by 10% in 2020–2021 as compared to 2019–2020. NFSA, National Food Security Act; PDS, public distribution system.

*Sources:* Commission for Agricultural Costs and Prices (2019a, b) and 13; Department of Food and Public Distribution (2020); Registrar General and Census Commissioner (2011); Ministry of Finance (2020).

The table shows excess stocks of rice and wheat following allocation of food grains through the universal PDS is much higher than the minimum buffer stock norms for both these crops.^24^ Our estimates show that the government will have to allocate 3.44% of the GDP to implement universal PDS in the rural areas and thereby enhance food security of the rural poor.

### Health provisions through the PDS

Initiation of economic activities in the rural areas—wage work and cultivation of agricultural crops—has the risk of jeopardizing health of workers who are participating in such activities since there is de-facto community transmission of Covid-19 in many parts of India. Hence, adequate health safety measures for workers are essential to initiate production-related activities in a sustainable manner. This assumes importance in view of decline in rural healthcare services following the pandemic due to near absence of ambulance services and lack of transportation, closure of private healthcare facilities, and suspension of public healthcare services to prevent the spread of disease as noted by Sundararaman and Ranjan (2020). In this context, initiation of production activities needs to be complemented with public provision of health kits through the public distribution system in rural areas. In this paper, we argue for universal provision of three-ply surgical masks (or other comparable masks) and hand sanitizers in rural areas. We also argue that this is the basic minimum that the state should provide for minimizing health risks of workers during the Covid-19 pandemic. Following the spread of the Covid-19 pandemic, there have been some initiatives related to health safety measures by the state (Ghosh, 2020; Ramakrishnan, 2020; The Hindu, 2020). We argue for extending these initiatives in the following ways:

- Universal provision of 16 three-ply masks and a pack of twenty-four 50-ml health sanitizer bottles in the rural areas for financial year of 2020–2021.
- Masks may be provided free of cost and sanitizers may be provided at a nominal price (of Rs 5 per 50-ml tube) through the PDS. Based on the initiatives by the Government of Kerala in producing hand sanitizers, priced at Rs 125 for 500 ml (Rs 12.5 for 50 ml), we argue that the government will have to pay a subsidy of Rs 7.5 per 50-ml tube of sanitizer if it is sold at Rs 5 through the PDS.

Table 7 sets out, in some detail, the required state provisioning of these basic health requirements for working families in rural areas. Government expenditure to the tune of almost Rs 10,087.2 crores would be required for providing basic health safety requirements for rural working families. Here, we are arguing for creating a flexible pool under the National Rural Health Mission for Covid-19 (see the union budget documents of the Ministry of Health and Family Welfare, 2020a). The share of this proposed expenditure in revised GDP of 2020–2021 is estimated at 0.05%.

**Table 7:** Health provision through the public distribution system in rural areas

Relevant indicators in the rural areas	
Total number of households in the rural areas (in crores)	23.1
Government spending per household (in Rs)	436
Total expenditure by the government for universal provisioning in the rural areas (in Rs crores)	10087.2
Share in health budget of 2020–2021 (%)	14.6
Share in GDP of 2020–2021 (%)	0.05

*Note*: Projected rural population in 2020 according to the Census of India 2011 database was 925,433,227.3 crores. Assuming average household size of four, total number of rural households in 2020 is 231,358,306.8 crores.

Price of each three-ply mask is Rs 16. This is as per the Government of India circular dated 24 March 2020.

Budget allocations for health includes allocations for the Department of Health Research, Department of Health and Family Welfare and the Ayurveda, Yoga and Naturopathy, Unani, Siddha and Homeopathy (AYUSH).

GDP in 2020–2021 has been estimated based on the assumption that there will be decline in nominal growth rate by 10% in 2020–2021 as compared to 2019–2020.

*Sources:* Registrar General and Census Commissioner (2011); Ministry of Consumer Affairs Food and Public Distribution (2020d); Ministry of Health and Family Welfare (2020a and 2020b); Ministry of Ayurveda, Yoga, Naturopathy, Unani, Siddha and Homeopathy (AYUSH (2020).

The different components of proposed government support have been listed in Table 8. We argue that this composite policy is required to not only deal with the aggravation of the agrarian crisis but also stimulate the economy, which was already reeling under the economic slowdown since 2018. Even though the government has claimed to allocate 10% of the GDP under the *Atmanirbhar Bharat* package, these claims have been at best viewed with skepticism (see Basole and Coutinho, 2020; Ghosh, 2020; Singh, 2020). In this paper, we argue that fiscal support by the Union government to the extent of 8.04%–10.3% of the GDP is required to implement the proposed composite policy response.

**Table 8:** Extent of government support for rejuvenating the economy

Nature of government support	Extent of government support (in Rs)
Wage subsidy	4702 crores
Fertilizer subsidy	11,000 crores
Credit subsidy	189,331.2 crores
Price support in the output market	89,476 crores
Extension of the MGNREGS	123,257 crores
	151,921 crores
	334,558 crores
	542,868 crores
Extension of the Garib Kalyan Rozgar Yojana	423,150 crores
Extension of the National Food Security Act, 2013	633,587 crores
Extension of health provisions	10,087.2 crores
Total government support	14.8 lakh crores–19 lakh crores
Share in GDP of 2020–2021	8.04%–10.3%

*Note*: GDP in 2020–2021 has been estimated based on the assumption that there will be decline in nominal growth rate by 10% in 2020–2021 as compared to 2019–2020. MGNREGS, Mahatma Gandhi National Rural Employment Scheme.

*Source:* As mentioned in Tables 1–7.

## Conclusion

The previous section shed light on the details of the proposed expenditure by the government, which is required to tackle the multipronged crisis that is confronting agrarian India. This would allow each of the six dimensions of the process of aggravation of the agrarian crisis in India on account of the Covid-19 pandemic, which were discussed in the earlier section, to be tackled in a preliminary way.

A limitation of our policy simulation exercise may arise if the actual allocations on various policy proposals turn out to be different from the estimated amounts or if the macroeconomic fundamentals like the GDP turn out to be different than expected. Another limitation is primarily on account of its being a transitional policy. If we assume that the transitional policy platform is implemented, it is likely that proposed policy initiatives such as wage subsidy, fertilizer subsidy, credit subsidy, and output price support are likely to be effectively implemented. The rural elite are likely to be disproportionate beneficiaries of these policies. However, any attempt to implement other proposed policy initiatives such as extension of employment guarantee, enhanced food security, and augmentation of rural public health, which directly empower the rural proletariat and poor peasants, are likely to be stymied to varying degrees by the contradictory alliance between the rural elite and the big bourgeoisie. It would be in the objective interest of the urban proletariat to strive for the realization of such a provisional policy platform not only because nonagricultural employment (and real wages) would increase as a result but also because many members of the former are rural-urban migrants with extant ties to the rural areas. The limitations of the proposed transitional policy platform that we have pointed out are not reasons to abandon the initiative. Instead, they are indicative of the fact that any such process would have to go further even in order to sustain such policies. We return to this point briefly in the last part of this section.

This composite proposal is eminently within the realm of the possible even in the conditions that obtain in India in the end of the second decade of the 21st century. However, any composite proposal such as this is not possible without there being a decisive break with the neoliberal project in India.^25^ The composite proposal advanced here would require a rise in government expenditure. If income and wealth taxes cannot be increased in the short run, then it would be necessary to increase the fiscal deficit. This would possibly result in a sovereign ratings downgrade by credit ratings agencies, which would in all likelihood be followed by capital flight. Therefore, such a composite policy response to the aggravation of India’s agrarian crisis on account of the Covid-19 pandemic would require the government to institute capital controls.^26^

Apart from this, an increase in government expenditure would induce increases in aggregate demand, output, and employment in the nonagricultural sectors too. This positive demand stimulus would be significant since the import intensity of production of both the nonlabor means of production employed in agriculture as well as the means of consumption that would be demanded by the rural poor would be relatively low. This increase in output would also increase tax revenue, which implies that the effective increase in the fiscal deficit would be lower than the increase in government expenditure. However as in the case of public provision of safety gear in the rural areas, a similar initiative would also be required in the urban areas to ensure that workers and petty producers in urban areas are not exposed to Covid-19 given that there is de-facto community transmission of Covid-19 in many parts of India.

However, such a composite policy response can only be a transitional one. A decisive break with the neoliberal project in India would require political mobilization by workers and peasants that may involve some of these policy proposals. But such mobilization may also go on to further demands in the agrarian setup, such as redistributive land reform, universal employment guarantee, agricultural minimum wage, enhanced public investment in agriculture, authentic expansion of cooperatives, and so on. Likewise, a transition to agro-ecological farming (based on cooperatives) as opposed to conventional farming (dominated by the rural rich) would constitute another break with the neoliberal project in India. Prospects of implementation of such a policy response would also involve a key role for state governments. It is also likely that the first possible steps in this direction are initiated by state governments, especially those that are constituted by struggles involving workers and peasants.

## Notes

1. According to Ramachandran (2011), “In Marxism, the term ‘agrarian question’ can be seen as having three broad aspects or component parts. The first concerns the nature, extent and degree of the development of capitalism in the countryside. The second aspect or component part of the agrarian question concerns the nature of the classes that arise on the basis of the development of capitalism in agriculture. This aspect addresses, for instance, questions of the characteristics of landlordism, of the forms of differentiation of the peasantry, the nature of moneylenders and merchant capitalists in the countryside, and of the socioeconomic characteristics of the rural labor force. The third aspect is concerned with class struggle: how and by means of what alliances are the classes that have been identified to be mobilized for a resolution of the agrarian question, that is, for a progressive transformation of production relations in agriculture and the socioeconomic conditions in the countryside?”

2. One issue, in this respect, which we hope to deal with in future work is the role of uneven development in the agrarian question in India. Mishra (2020) is a recent contribution that deals with some aspects of this issue.

3. The agrarian experience of China during the Covid-19 pandemic has been examined in Fan (2020). See Patnaik (2008) who sets out in detail the processes of agrarian crisis in India during the neoliberal period.

4. For some classes, particularly the rural poor, one or more of these components may not involve exchange and therefore imputed magnitudes may need to be employed.

5. The transitional policy platform that is discussed in this section does not involve targeting since historical experience indicates that targeting is not optimal due to factors connected to political economy that is manifested as inadequacies in “state capacity.” In other words, targeting typically leads to exclusion of some sections of the working poor.

6. There have been different ways adopted to examine the differentiation of the peasantry, but in this paper we do not necessarily advocate any particular method.

7. The National Sample Survey Office (2014a) is one such database that estimates the number of households in various sections of the peasantry and other labor hiring classes that were involved in crop production and expenses on labor. This is crucial for estimating wage subsidy to households involved in crop production. The survey of agricultural households was done in 2012–13.

8. As on 4 December 2020, the conversion rate was 1 US Dollar = Rs 73.7. This is as per the data provided by Financial Benchmarks India Private Limited (2020). The Reserve Bank of India had mentioned that since 2018, computation and dissemination of conversion rate is provided by the FBIL (https://www.rbi.org.in/Scripts/BS_DisplayReferenceRate.aspx).

9. Financial costs are defined as actual costs of raising financial resources; transaction costs incorporate salaries of staff, rent, travel and other organizational costs for undertaking daily institutional operations and risk costs consist of actual write-offs and reserves created for bad debt.

10. RBI has illustrated that this is the credit limit for a small farmer who operates on two acres of land and raises multiple crops in a year. See RBI-Revised Scheme for Issue of KCC (available at https://rbidocs.rbi.org.in/rdocs/content/pdfs/CRB5100512AN.pdf).

11. In this analysis, even though the quantum of credit subsidy, 10.5%, is perhaps on the higher side, if one estimates credit subsidy to be provided by the commercial banks as the difference between lending and deposit rates (as seen in Reserve Bank of India (2010); bankbazaar.com, 2020b). Yet, the credit limit (Rs. 1.33 lakh), based on the RBI’s illustration is the minimum level of credit support for farmers who operate on two acres (0.8 hectares) of land. In this sense, we are arguing for a fairly limited government intervention in the credit market in rural areas.

12. This is based on the recommendations of the Department of Food and Public Distribution (2002) and proposals of the National Commission on Farmers (Swaminathan, 2016). According to the Directorate of Economics and Statistics (2000), A_2_ + FL includes value of hired human labor + value of hired bullock labor + value of owned bullock labor + value of owned machinery labor + hired machinery charges + value of seed + value of insecticides and pesticides + value of manure + value of fertilizer + depreciation of implements and farm buildings + irrigation charges + revenue, cesses and other taxes + interest on working capital + miscellaneous expenses + rent paid for leased in land + imputed value of family labor (based on market wage rate or statutory wage rate whichever is higher). C_2_ includes value of hired human labor + value of hired bullock labor + value of owned bullock labor + value of owned machinery labor + hired machinery charges + value of seed + value of insecticides and pesticides + value of manure + value of fertilizer + depreciation of implements and farm buildings + irrigation charges + land revenue, cesses and other taxes + interest on working capital + miscellaneous expenses + interest on value of owned fixed capital assets (excluding land) + rental value of owned land and rent paid for leased-in land + imputed value of family labor.

13.
Commission for Agricultural Costs and Prices, 2019a, Commission for Agricultural Costs and Prices, 2019b; Commission for Agricultural Costs and Prices (2019a). In this analysis, only one variety, each of paddy, *jowar*, and cotton, has been considered. In all, CACP recommends MSP for 24 crops.

14. This shows that wages are important sources of income for poor peasants and a section of the middle peasants and for those who do not have access to land (rural proletariat). See National Sample Survey Office (2014a), which goes on to define an agricultural household as “a household receiving some value of produce from agriculture activities (e.g. cultivation of field crops, horticultural crops, fodder crops, plantation, animal husbandry, poultry, fishery, piggery, bee-keeping, vermiculture, sericulture, etc.) during last 365 days.”

15. However, some economists have also argued that proliferation of informal sector employment, primarily in big and some other urban centers in India, does not reflect dynamism of the nonfarm sector (e.g. Basole and Basu, 2011b; Sanyal and Bhattacharyya, 2009). If that was the case, then formal sector employment would have expanded in these areas.

16. Expansion of state intervention in the rural labor market in the ambit of the democratic movement will erode the bargaining power of rural rich. Besides, by making a dent in the interlinkages across rural markets that serves the interests of rural rich, such intervention will lead to some enhancement of freedom of the workers many of whom belong to the oppressed strata in India. Recent reports seem to suggest that in the post-Covid-19 period, with lockdowns and restrictions on labor movement, there were attempts by sections of the rural rich (who are also the mainstay of social oppression in rural areas) in certain parts of India, to reduce wages of rural workers (see Tur, 2020: Bansal, 2020).

17.
Gidwani (2018) examines the consequences of informality on the relation between the active and reserve armies of labor.

18. See Kandikuppa and Gupta (2020) for a detailed analysis of the structural factors that had led to the crisis that the migrant workers have been facing with the onset of neoliberal policies in India.

19. This would be an authentic addition to the other existing schemes that have been announced by the Union government, following the emergence of Covid-19 pandemic, if aggregate public expenditure on all such schemes increases by the amount that has been announced.

20. This is almost eight times higher than what the office of the Chief Labor Commissioner (central) had mentioned in June 2020. See Chief Labour Commissioner (Central) (2020) available at https://clc.gov.in/clc/node/647 for detailed list.

21. But the Indian government, which is an integral part of and promoter of the neoliberal project in India, is not keen to use its food stocks that are held through its nodal agency, the Food Corporation of India for this purpose. Paradoxically, Rawal et al. (2020) noted that fiscal orthodoxy (which is definitive of the neoliberal project as long as the interests of finance capital do not require government spending) of the Indian government had resulted in steady increases in excess stocks (above stocking norms) of the FCI since October 2018 and by 1 May 2020, the government had excess stocks of almost 67 million tons of food grains. Earlier, Patnaik (2003) argued that burgeoning food stocks with the government reflects erosion of purchasing power of the working poor and therefore reduced absorption of food grains among vast sections of the population. Indeed, job losses have compelled the working poor to the margin of subsistence and some below it due to the rapid spread of the Covid-19 pandemic. Loss of work of large sections of the rural poor (from rural proletariat, poor peasant, or some middle peasant households)—many of whom are migrant workers associated with the informal sector—have resulted in a further increase in food insecurity.

22. According to the NFSA, AAY households are entitled to receive 35 kg of food grains per family per month; food grains like rice, wheat, and coarse grains are to be made available at Rs 3, 2, and 1 respectively. The nuances and political economy of the food security act was discussed in detail by Ahmed and Chatterjee (2013).

23. According to the FAO (2003), fat content in wheat, rice, other cereals, and barley are equal at 8.37 Kcal/gram. Protein content has been estimated at rice (white)—3.82 Kcal/gram, wheat (97%–100% extraction)—3.59 Kcal/gram, other cereals (refined)—3.87 Kcal/gram, and barley, pearled—3.55 Kcal/gram. Corresponding figures for carbohydrate are 4.16, 3.78, 4.12, and 3.95. In this paper we have considered the fat content only for our analysis.

24. As per the Department of Food and Public Distribution, Government of India, buffer stock norms of rice are 13.6 and 10.3 million tons in July and October, respectively; corresponding figures for wheat are 27.6 and 20.5 million tons.

25. The recent move by the central government of India in fact amounts to a consolidation of the agrarian dimension of the neoliberal project. Details of these changes can be seen in the Union Cabinet Press Release (2020).

26. Capital controls involve restrictions on cross-border movement of finance capital.

27. This pattern of technical change is likely in a social formation dominated by the capitalist mode of production. Therein, a rise in the technical composition of capital (a rise in the means of labor used per unit of output) may decrease the labor required per unit of output or the land required per unit of output.
